# Interactions between Co-Habitating fungi Elicit Synthesis of Taxol from an Endophytic Fungus in Host *Taxus* Plants

**DOI:** 10.3389/fmicb.2013.00003

**Published:** 2013-01-22

**Authors:** Sameh S. M. Soliman, Manish N. Raizada

**Affiliations:** ^1^Department of Plant Agriculture, University of GuelphGuelph, ON, Canada; ^2^Faculty of Pharmacy, Zagazig UniversityZagazig, Egypt

**Keywords:** *Paraconiothyrium*, fungus, Taxol, *Taxus*, elicitor, endophyte

## Abstract

Within a plant, there can exist an ecosystem of pathogens and endophytes, the latter described as bacterial and fungal inhabitants that thrive without causing disease to the host. Interactions between microbial inhabitants represent a novel area of study for natural products research. Here we analyzed the interactions between the fungal endophytes of *Taxus* (yew) trees. Fungal endophytes of *Taxus* have been proposed to produce the terpenoid secondary metabolite, Taxol, an anti-cancer drug. It is widely reported that plant extracts stimulate endophytic fungal Taxol production, but the underlying mechanism is not understood. Here, *Taxus* bark extracts stimulated fungal Taxol production 30-fold compared to a 10-fold induction with wood extracts. However, candidate plant-derived defense compounds (i.e., salicylic acid, benzoic acid) were found to act only as modest elicitors of fungal Taxol production from the endophytic fungus *Paraconiothyrium* SSM001, consistent with previous studies. We hypothesized the *Taxus* plant extracts may contain elicitors derived from other microbes inhabiting these tissues. We investigated the effects of co-culturing SSM001 with other fungi observed to inhabit *Taxus* bark, but not wood. Surprisingly, co-culture of SSM001 with a bark fungus (*Alternaria*) caused a ∼threefold increase in Taxol production. When SSM001 was pyramided with both the *Alternaria* endophyte along with another fungus (*Phomopsis*) observed to inhabit *Taxus*, there was an ∼eightfold increase in fungal Taxol production from SSM001. These results suggest that resident fungi within a host plant interact with one another to stimulate Taxol biosynthesis, either directly or through their metabolites. More generally, our results suggest that endophyte secondary metabolism should be studied in the context of its native ecosystem.

## Introduction

Taxol is a diterpenoid anti-cancer drug harvested from *Taxus* (yew) trees, worth more than $2 billion USD annually to the pharmaceutical industry (Han et al., 2007; Roberts, 2007). Taxol is found in very low concentrations in yew trees prompting the search for alternative sources (Stierle et al., 1995). Endophytes are microorganisms (fungi and bacteria) that live within a plant without causing disease symptoms. Nearly 20 years ago, it was first reported that Taxol could be synthesized by a fungal endophyte, *Taxomyces andreanae*, discovered living within the inner tissues of *Taxus brevifolia* (Stierle et al., 1993). The authenticity of fungal Taxol was confirmed using mass spectrometry, NMR, and recognition by a plant Taxol monoclonal antibody (Li et al., 1996; Moon-Jong et al., 1999). Some studies have however questioned the authenticity of fungal-derived Taxol and some controversy remains whether plant-derived compounds are required for its synthesis (Staniek et al., 2009). Several studies have shown, however, that fungal Taxol is produced independently of the plant following *in vitro* culturing (Li et al., 1996; Strobel et al., 1996b; Guo et al., 2006). The contradictory evidence that fungi can produce authentic Taxol and can do so autonomously may be due to variation between fungal isolates in previous studies: fungi can be polynucleate and genetically unstable. Furthermore, fungal age, storage conditions, and environment may have varied between published studies conducted in different labs. Multiple labs now claim that at least 18 different fungal genera produce Taxol (Zhou et al., 2010).

We recently cultured a novel Taxol-producing fungus living inside *T. x media* wood and identified it as *Paraconiothyrium* strain SSM001 (Soliman et al., 2011). We demonstrated that SSM001 could produce Taxol independently of plant tissues following two cycles of *in vitro* hyphal tip transfer and inoculation into liquid media where the fungus grew >1000-fold prior to peak Taxol production, all in the absence of any plant tissues or extracts (Soliman et al., 2011). The fungus was localized to the living, nutrient-rich vascular tissues that radially traverse wood, known as wood medullary rays.

In general from both ecological and economic viewpoints, a microbial source of Taxol would be more useful than yew plants. However, though initially an exciting discovery, the amount of Taxol produced by the initial Taxol-producing fungus, *T. andreanae*, was found to be too low for commercial production, in the range of only micrograms per liter in liquid culture (Stierle et al., 1993; Strobel, 2003; Strobel and Daisy, 2003). Other genera of Taxol-producing fungi were subsequently found to produce similarly low levels of Taxol, including, for example: *Pestalotia heterocornis*, which was isolated from soil collected in a yew forest (Noh et al., 1999); *Pestalotiopsis microspora*, a common fungus present in tropical and semitropical plants, and a widespread saprophyte on bark and decaying plant material (Metz et al., 2000); and BT2, an endophytic fungus isolated from *T. chinensis var. mairei* plants (Guo et al., 2006).

Several attempts have been made to enhance the production of fungal Taxol. The addition of phenylalanine, a precursor of the Taxol side-chain, increased Taxol production from plants (Veeresham et al., 2003) and fungi (Strobel et al., 1996a). Taxol production in *P. microspora* increased when phosphate was decreased and sodium benzoate was added (Li et al., 1998a). In addition, sterol biosynthesis inhibitors, such as tebuconazole and triadimefon, have been shown to increase fungal Taxol yields (Li et al., 1998a). In addition to chemical elicitors, mutagenesis of *Fusarium maire* protoplasts by UV radiation and diethyl sulfate (DES) treatment resulted in a mutant with increased production of Taxol, with a final yield of 225.2 μg/L (Xu et al., 2006). Similarly, a combination of UV irradiation, ethyl methyl sulfonate (EMS), ^60^Co, and nitrosoguanidine (NTG) mutagenesis treatments of *Nodulisporium sylviform*, improved Taxol production from 51–126 to 314 μg/L, resulting in strain NCEU-1 (Zhao et al., 2008; Zhou et al., 2010). Taxol output by NCEU-1 was further improved to 418 μg/L by UV irradiation and LiCl treatment (Zhao et al., 2008; Zhou et al., 2010). Independently, four cycles of random genome shuffling of NCEU-1 by protoplast fusion increased Taxol output from 314 to 516 μg/L (Zhao et al., 2008; Zhou et al., 2010).

Despite the above attempts, the largest increase ever observed in fungal Taxol production was also part of the first attempt to improve it, namely the addition of yew needle broth, which reportedly increased fungal Taxol production by 100-fold (Stierle et al., 1995). More recently, co-culture of a Taxol-producing *Fusarium* with *Taxus* suspension cells resulted in a 38-fold increase in Taxol production compared to *Fusarium* alone (Li et al., 2009). Despite these promising results, attempts have not been successful in discovering the underlying plant-derived elicitor(s), which may include precursors of the fungal Taxol pathway, of which only phenylalanine has been tested as noted above (Stierle et al., 1993; Strobel et al., 1996a). Clues for fungal Taxol elicitors may come from the compounds found in the nutrient-rich vascular tissue habitat of endophytic fungi, which could include various nutrient sources, other chemical factors such as plant hormones, and plant defense compounds produced in response to fungal invasion. Other candidate elicitors might be derived from organisms that share the habitat of these endophytes including other fungi.

The objective of this study was to identify novel elicitors of Taxol synthesis from the fungal endophyte *Paraconiothyrium* SSM001 based on compounds or organisms expected to exist in the native habitat inside *Taxus* plants. Our most surprising result was that interactions between co-habitating fungi can stimulate endophytic fungal Taxol production.

## Materials and Methods

### Strains

The fungal strain described in this paper, *Paraconiothyrium* SSM001, has been deposited at ATCC (Accession # ATCC MYA-4697).

### Chemicals

The following reagents were from Sigma (USA), including taxane standards: Taxol (# T7402), baccatin III (# B8154), and cephalomannine (# C4991); fungal nutrient media: yeast-peptone-dextrose (YPD; # Y1375) and potato-dextrose-agar (PDA; #70139); and candidate elicitors: benzoic acid (#242381), salicylic acid (#S0875), methyl jasmonate (#392707), indole acetic acid (IAA; #15148), and resveratrol (#R5010). Strigolactone (#GR24) was from Dr. B. Zwanenburg (Radboud University, Nijmegen, Netherlands). All solvents used for extraction, TLC, and HPLC, were HPLC grade and obtained from Fisher Scientific.

### Isolation of endophytic fungi

Endophytic fungi were cultured from old branches of *T. x media* trees grown on the Main Campus and Arboretum of the University of Guelph (Guelph, ON, Canada). Fungi were isolated from fresh plant tissues following harvesting. *Taxus* branches were cut into 1 cm long × 0.5 cm diameter sections and sterilized as follows: 2.5% sodium hypochlorite solution for 10 min; 70% ethanol for 5 min; washed in sterile double distilled water three times. The outer bark was stripped away and the inner tissues were further sterilized using 70% ethanol for 5 min followed by flaming and washing three times with sterile double distilled water. Each piece of tissue was then cut into smaller pieces (2 mm × 5 mm) and cultured on PDA media in Petri plates at 25°C in the dark until initial fungal growth was observed. Hyphal tips were then successively transferred twice onto fresh PDA media to ensure fungal culture purity (Li et al., 1996).

### Fungal genotyping

For consistency between experiments, every fungal liquid culture was genotyped by PCR and DNA sequencing of the internal transcribed spacer regions (ITS) of 18S rDNA (Perez-Vera et al., 2005) to confirm both strain identity and purity. The fungal mycelia were lysed by mortar and pestle with liquid nitrogen and then genomic DNA was isolated using the Plant DNeasy Kit (Qiagen, Germany) with additional adsorption of the DNA onto a silica membrane, washing, and centrifugation as previously described (Haugland et al., 2002). Fifty nanograms of the DNeasy-purified genomic DNA were added into a 20-μl PCR reaction, with 1X buffer (Green GoTaq Flexi Buffer, Promega, USA, # M8911), 1.5 mM MgCl_2_, 0.2 mM dNTPs, 0.5 U *Taq* DNA polymerase (New England BioLabs, USA), and 0.2 μM of each universal primer set specific for fungal ITS1 and ITS4 (Perez-Vera et al., 2005). The following conditions were used for PCR amplification: 94°C for 2 min, followed by 35 cycles of: 94°C for 45 s, 45°C for 1 min, and 72°C for 2 min; with a final extension cycle of 4 min at 72°C. The amplicon was purified using an Illustra GFX 96 PCR Purification kit (GE Healthcare, USA), cloned, and sequenced. Each amplicon was then aligned with the sequence of strain SSM001 using Align-BLAST software.

### Terminal restriction fragment length polymorphism analysis

For *in planta* fungal DNA fingerprinting, genomic DNA from *Taxus* and SSM001 (positive control) were used to amplify 18S rDNA using fluorescent-labeled fungal 18S primers. Amplicons were digested with *Hae*III or *Hhe*I, precipitated, mixed with 8 μl GeneScan 500 TAMRA size standards (PE Applied Biosystems, UK), denatured, and loaded onto a ABI Prism310 DNA Sequencer (PE Applied Biosystems, Canada). Fragment data was collected with ABI Prism310 (v.2.0) and analyzed with Peak scanner (v.1.0).

### Identification and quantification of fungal taxol in liquid culture

Fungal tips (∼10 mg) from 2-week-old pure PDA plate cultures were cultured into 1 L Erlenmeyer flasks containing 500 ml of liquid YPD media and incubated for 3 weeks in the dark at room temperature. The mycelia were filtered from the liquid media using cheesecloth. The filtered liquid media was extracted three times with 50 ml of chloroform/methanol (9:1 v/v). The organic layers were separated, collected, and washed using distilled water and then evaporated at 45°C using a Rotavapor until fully dried.

For identification of fungal Taxol by TLC, the residue from each extract was dissolved in 30 μl of methanol, and 8 μl applied onto silica gel plates (10 cm × 20 cm, Fisher Scientific #4861-320 and #11028) alongside the taxane standards, Taxol, cephalomannine, and baccatin III, each at a concentration of 25 μg/ml. TLC plates were then developed in a solvent system (chloroform/methanol, 5.0:0.5). For compound visualization, TLC plates were dipped in 0.5% vanillin/sulfuric acid reagent for 1 min. For HPLC identification of Taxol, fungal Taxol with the same *R*_f_ value of standard Taxol was purified from several TLC plates prior to LC-MS injection. A LCQ DECA ion trap LC-MS instrument (Thermo Finnigan, USA) was used, equipped with a Finnigan SpectraSystem UV6000LP UV detector and a RESTEK Ultra Phenyl column (250 mm × 2.1 mm, 5 μm). The binary mobile phase consisted of solvent A (0.1% formic acid) and solvent B (acetonitrile). A gradient program was used to run the analyzed samples as follows: isocratically at 80% A for 1 min, 80-30% A for 24 min, 30-80% A for 5 min, and isocratically at 80% A for 5 min. The flow rate was 0.4 ml/min. Taxol was measured using the UV absorbance at 233 nm. Taxane standards were run in parallel. The electrospray (ESI) positive ion mode was used for ion detection. The system was operated as follows: shear gas and auxiliary flow rates were set at 96 and 12 (arbitrary units); voltage setting on the capillary; tube lens offset; multipole one offset; multipole two offset; lens and entrance lens were set at 32.50, 55.00, −4.40, −8.00, −14.00, and −58.00 V, respectively; the capillary temperature was set at 350°C; and the ion spray voltage was controlled at 5 kV.

Fungal Taxol was also identified and quantified using a competitive immunoassay procedure, which employed a kit (#TA02, Hawaii Biotechnology Group, Inc., USA) in conjunction with a commercial Taxol monoclonal antibody (#SC-69899, Santa Cruz Biotechnology Inc., USA). Manufacturer’s instructions were followed. In order to buffer against interfering fungal metabolites in the fungal extract, each immunoassay read was compared to a standard curve generated each time consisting of Taxol standard added into fungal extract from non-Taxol-producing *Fusarium*. The specificity of the Taxol immunoassay was confirmed by testing different concentrations of diverse taxanes (baccatin III and cephalomannine) either alone or added to wells containing Taxol. HPLC of total fungal *Paraconiothyrium* extract was compared to diverse taxane standards to determine if they were present.

Additional details were noted in a previous paper (Soliman et al., 2011).

All Taxol concentrations were normalized to dry weight of the fungal mycelia, because we were concerned that some of the elicitors could affect the growth of the fungal mycelia, and also because we noticed flask-to-flask variation in the growth of the mycelia.

### Plant-fungal light microscopy

For *in vivo* light microscopy, sterilized longitudinal and transverse thin wood sections were cultured on PDA media for 12 h followed by fixation and staining for 1 week in Trypan blue (dissolved in ethanol).

Fungal spore examination was performed by stripping off the bark and wood pieces, followed by attaching the inner side of the bark and wood to two-sided tape fixed on a microscopic slide. The tissues were pressed for a few seconds then removed, and the tape was stained with Trypan blue prior to light microscopy (Deckert et al., 2001).

### Post-inoculation treatments of fungal liquid cultures

Fungal liquid cultures, incubated in the dark at 25°C, with shaking at 100 rpm (inoculation conditions as described above), were treated with either plant or fungal tissues or chemicals. For testing the effects of plant tissues, the following was added at 10 days post-inoculation: *T. x media* wood pieces (0.5 g) and outer bark pieces (0.5 g) or their alcohol extracts (5 ml); *Pinus strobus* wood pieces (0.5 g) and outer bark pieces (0.5 g) or their alcohol extracts (5 ml); and *Taxus x media* callus suspension culture (30 ppm, see below). For studying the effects of non-*Paraconiothyrium* fungi, we isolated *Alternaria* and *Phomopsis* from *T. x media* outer bark and needles, respectively and then added 0.2 g enclosed within Miracloth (Calbiochem-Novabiochem Corporation, La Jolla, CA, USA, #475855) to a 10-day old *Paraconiothyrium* liquid culture. For chemical treatments, the following supplements were added at 10 days post-inoculation: benzoic acid (0.01 mM), salicylic acid (0.28 mM), methyl jasmonate (200 mM), IAA (1.01, 2.02, and 3.04 mM), strigolactone (0.1, 1.0, and 10 ppm), and resveratrol (0.876 mM). Each chemical was either dissolved or suspended in 1 ml of DMSO with 4 ml of 70% ethanol as solvent. As a control, an equal volume of solvent only was added to a parallel liquid culture. All cultures continued their incubation at 25°C in the dark, shaking at 100 rpm, for an additional 14 days, after which fungal Taxol was extracted as described above. The results shown represent the mean of three independent experiments (three separate flasks). Statistical comparison of means, along with all raw data, is presented in Table 1 with each statistical test noted, conducted using In Stat 3.0 (GraphPad Software, USA).

**Table 1:** **Raw data of biological replicates and statistical analysis**.

Experiment description*	Figure number	Control data** (μg/g)	Treatment details	Treatment data (μg/g)	Stat test	Significance
Effect of plant tissues on fungal taxol production	Figure 1B	3.50	*Taxus* wood (0.5 g)	2.99	One-way analysis of variance (ANOVA)	*P* value is 0.0086
		3.00		4.24	Comparison:	
		2.40		5.00	Solvent vs. *Taxus* wood	*P* > 0.05
			*Taxus* bark (0.5 g)	7.97	Solvent vs. *Taxus* bark	*P* < 0.01
				5.55	Solvent vs. Pine wood	*P* > 0.05
				5.63	Solvent vs. Pine bark	*P* > 0.05
			Pine wood (0.5 g)	3.87	*Taxus* wood vs. *Taxus* bark	*P* > 0.05
				4.42	*Taxus* wood vs. Pine wood	*P* > 0.05
				3.52	*Taxus* wood vs. Pine bark	*P* > 0.05
			Pine bark (0.5 g)	5.92	*Taxus* bark vs. Pine wood	*P* > 0.05
				4.30	*Taxus* bark vs. Pine bark	*P* > 0.05
				5.65	Pine wood vs. Pine bark	*P* > 0.05
Effect of plant tissue extracts on fungal taxol production	Figure 1C		*Taxus* wood extract (5 ml of 0.1 g tissue/ml)	31.11	One-way analysis of variance (ANOVA)	*P* value is < 0.0001
				22.12	Comparison:	
				35.92	Solvent vs. *Taxus* wood extract	*P* < 0.001
			*Taxus* bark extract (5 ml of 0.1 g tissue/ml)	89.20	Solvent vs. *Taxus* bark extract	*P* < 0.001
				96.01	Solvent vs. Pine wood extract	*P* < 0.01
				85.20	Solvent vs. Pine bark extract	*P* < 0.001
			Pine wood extract (5 ml of 0.1 g tissue/ml)	20.93	*Taxus* wood extract vs. *Taxus* bark extract	*P* < 0.001
				28.22	*Taxus* wood extract vs. Pine wood extract	*P* > 0.05
				19.42	*Taxus* wood extract vs. Pine bark extract	*P* < 0.001
			Pine bark extract (5 ml of 0.1 g tissue/ml)	56.29	*Taxus* bark extract vs. Pine wood extract	*P* < 0.001
				60.01	*Taxus* bark extract vs. Pine bark extract	*P* < 0.001
				68.01	Pine wood extract vs. Pine bark extract	*P* < 0.001
Effect of *Taxus* tissue culture on fungal taxol production	Figure 1D		Addition of *Taxus* suspension culture (30 ppm)	2.80	Unpaired *t* test *t* = 1.335 with 4 df	*P* value is 0.2529
				2.50		
				2.07		
Effect of resveratrol on fungal taxol production	Figure 1E		Effect of resveratrol (0.876 mM)	0.90	Unpaired *t* test *t* = 5.228 with 4 df	*P* value is 0.0064
				1.42		
				0.90		
Effect of salicylic acid and methyl jasmonate on fungal taxol production	Figure 1F		Salicylic acid (0.28 mM)	6.91	One-way analysis of variance (ANOVA)	*P* value is 0.0013
				5.70	Comparison:	
				5.21		
			Methyl jasmonate (200 mM)	1.84	Solvent vs. salicylic acid	*P* < 0.01
				2.21	Solvent vs. methyl jasmonate	*P* > 0.05
				2.92	Salicylic acid vs. methyl jasmonate	*P* < 0.01
Effect of benzoic acid on fungal taxol production	Figure 1G		Benzoic acid (0.01 mM)	5.10	Unpaired *t* test *t* = 6.128 with 4 df	*P* value is 0.0036
				4.90		
				5.45		
Effect of strigolactone on fungal taxol production	Figure 1H		Strigolactone 0.1 ppm	2.21	One-way analysis of variance (ANOVA)	*P* value is < 0.0001
				1.90	Comparison:	
				1.84		
			Strigolactone 1 ppm	1.19	Solvent vs. strigo 0.1 ppm	*P* < 0.05
				1.01	Solvent vs. strigo 1 ppm	*P* < 0.001
				0.92	Solvent vs. strigo 10 ppm	*P* < 0.001
			Strigolactone 10 ppm	0.84	Strigo 0.1 ppm vs. strigo 1 ppm	*P* < 0.05
				0.54	Strigo 0.1 ppm vs. strigo 10 ppm	*P* < 0.01
				0.33	Strigo 1 ppm vs. strigo 10 ppm	*P* > 0.05
Effect of IAA on fungal taxol production	Figure 1I		IAA 1 mM	0.00	One-way analysis of variance (ANOVA)	*P* value is < 0.0001
				0.10	Comparison:	
				0.10		
			IAA 2 mM	0.00	Solvent vs. IAA 1 mM	*P* < 0.001
				0.05	Solvent vs. IAA 2 mM	*P* < 0.001
				0.09	Solvent vs. IAA 3 mM	*P* < 0.001
			IAA 3 mM	0.00	IAA 1 mM vs. IAA 2 mM	*P* > 0.05
				0.04	IAA 1 mM vs. IAA 3 mM	*P* > 0.05
				0.08	IAA 2 mM vs. IAA 3 mM	*P* > 0.05
Effect of *Alternaria* on fungal taxol production	Figure 2H		*Alternaria* (0.2 g into 500 ml)	9.80	Unpaired *t* test *t* = 4.745 with 4 df	*P* value is 0.0090
				6.21		
				8.50		
Effect of *Phomopsis* on fungal taxol production	Figure 3C		*Phomopsis* (0.2 g into 500 ml)	10.20	Unpaired *t* test *t* = 5.146 with 4 df	*P* value is 0.0068
				14.30		
				9.15		
Effect of two fungal cultures on fungal taxol production	Figure 4B		*Alternaria* + *Phomopsis* (0.2 g each into 500 ml)	20.12	Unpaired *t* test *t* = 11.010 with 4 df	*P* value is 0.0004
				26.30		
				22.65		

*Taxol was quantified using the Taxol immunoassay (see Materials and Methods) using one fungal isolate of *Paraconiothyrium* SSM001. Each replicate shown represents a pool of 500 ml liquid fungal culture.

**All plant tissue extracts and chemical elicitors were either dissolved or suspended in ethanol:water (1:1).

### Callus and suspension culture protocol and taxane analysis

Needles and very young stem sections of *T. x media* were surface sterilized with 3% Clorox solution containing two drops of Tween 20 for 20 min with shaking. The sterilized explants were then dipped into 70% ethanol for ∼5 min and washed five times with sterile ddH_2_O. The tissues were then cut into small explants (0.5–1 cm^2^) prior to being placed onto B5CA culture media. B5CA consists of Gamborg’s B5 medium (Gamborg et al., 1968) supplemented with 0.2% casamino acids (CA), 1% sucrose, 1 mg/L 2,4-D, 0.75% agar, with pH adjusted to 5.5 prior to autoclaving (Gibson et al., 1993). Cultures were initiated in the dark at 25°C and subcultured at 1–2 months intervals.

Suspension cultures were initiated from 2-month-old calli by placing ∼2 g of tissue in 1 L Erlenmeyer flasks containing 100 ml B5CA media at 25°C in dark with shaking at 100 rpm for 2 weeks. The suspension cultures were continually grown for another 1 month. For taxane analysis, suspension cultures were filtered and the liquid media were extracted with 50 ml chloroform: methanol (10:1) three times. The filtered, washed organic layer was then evaporated at 45°C under reduced pressure. The residue was then subjected to LC-MS analysis against standards Taxol and baccatin III.

### Real-time qPCR

RNA was isolated and PCR reaction efficiencies were determined by a series of 10-fold dilutions using fungal 18S rRNA specific primers (Fang and Bidochka, 2006), 18S rRNA-RtF (5′-GGCATCAGTATTCAGTTGTC-3′), and 18S rRNA-RtR (5′-GTTAAGACTACGACGGTATC-3′). Amplification conditions were as follows: 95°C for 10 min, followed by 40 cycles of: denaturation, 95°C for 15 s; annealing 56.6°C for 30 s; extension at 72°C for 1 min. The specificity of the reaction was shown by the detection of the *T*_m_s of the amplification products immediately after the last reaction cycle. Results were analyzed with the melting curve StepOne analysis software (Applied BioSystems; Soliman et al., 2011). The relative expression ratio of the fungal gene was analyzed based on real-time PCR efficiency and the crossing point differences of the samples vs. the plant 18S rRNA (Tax 18S), Tax18SF2 (5′-TTTTCCCTTTGCAATGCC-3′), and Tax18SR2 (5′-TCGCCCTTGTAATAACCCG-3′). The results were verified using REST (Relative Expression Software Tool) as previously described (Soliman et al., 2011).

## Results

### Plant bark and wood extracts elicit fungal taxol biosynthesis

**Figure 1:**
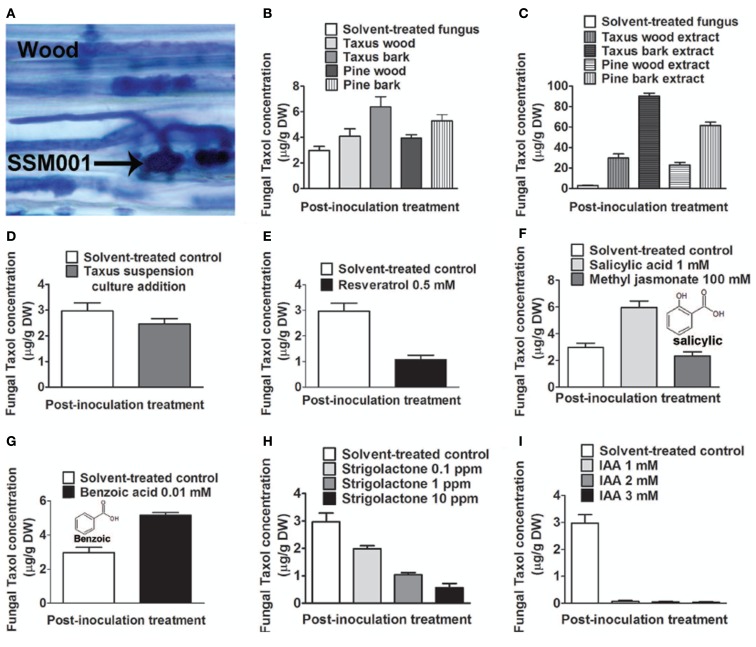
**Effect of plant extracts or candidate plant compounds, based on the habitat of *Paraconiothyrium* SSM001, on fungal Taxol production at 3 weeks following inoculation in fungal liquid culture**. **(A)** Trypan blue-stained SSM001 from 12-h-old cultured *Taxus x media* wood sections showing SSM001 localization to the wood vascular cells (wood medullary rays). **(B)** Effect of added *Taxus* and *Pinus* tissues. **(C)** Effect of added *Taxus* and *Pinus* alcohol extracts. **(D)** Effect of added taxane-producing *Taxus* suspension culture cells to test whether *Taxus* contributes precursors for fungal Taxol biosynthesis. **(E)** Effect of the antimicrobial phytoalexin, resveratrol. **(F)** Effect of plant defense hormones. **(G)** Effect of benzoic acid, a derivative related to salicylic acid. **(H)** Effect of strigolactone, a plant hormone that stimulates fungal germination and penetration, and which is localized to vascular cells. **(I)** Effect of indole acetic acid (IAA), a plant growth hormone important in vascular tissue biogenesis.

Taxol-producing strain *Paraconiothyrium* SSM001 was found to be localized within *T. x media* to the living, nutrient-rich vascular tissues that traverse dead plant wood, known as medullary rays (Figure 1A). We thus hypothesized that wood and/or bark may act as an elicitor of fungal Taxol production. We tested the effects of *T. x media* wood and bark pieces, and their respective alcoholic extracts, on fungal Taxol production. To exclude the possibility that plant taxanes may be directly contributing to any apparent increase in fungal Taxol, pine (*P. strobus*) was used as a control. Incubation of fungal cultures with yew bark pieces caused a modest (∼twofold) but significant (*P* < 0.01) increase in fungal Taxol production (Figure 1B; Table 1), whereas the corresponding ethanol extracts from 500 mg of plant tissue into 500 ml of fungal liquid culture caused dramatic increases in fungal Taxol yield (yew wood extract, 10-fold increase; yew bark extract, 30-fold; pine wood extract, eightfold; pine bark extract, 20-fold; all *P* < 0.01; Figure 1C; Table 1). Because pine extracts also caused such a dramatic increase in fungal Taxol yield, this strongly suggested the increase was due to either an early precursor in the Taxol pathway or an elicitor signal. To distinguish between these two hypotheses, we applied 30 ppm of a yew callus suspension culture that showed taxane-producing activity (data not shown). The *Taxus* callus suspension culture did not increase fungal Taxol production (Figure 1D; *P* = 0.25, Table 1), thus excluding the precursor incorporation hypothesis.

We then took a candidate approach to test for potential plant-derived elicitors of fungal Taxol biosynthesis. Since Taxol has anti-fungicidal properties and Taxol biosynthesis is stimulated by pathogenic fungi (Wang et al., 2000; Strobel, 2002, 2003; Strobel and Daisy, 2003), we hypothesized that plant defense compounds synthesized in response to pathogens might stimulate fungal Taxol biosynthesis. First we tested resveratrol, an antimicrobial phytoalexin derived from the phenylpropanoid pathway, shown to be induced in pine bark in response to pathogenic fungi (Evensen et al., 2000). However, resveratrol was observed to cause a dramatic and significant (*P* < 0.01) decline rather than increase in fungal Taxol production (Figure 1E; Table 1).

Salicylic acid (Pastírová et al., 2004; Jacek et al., 2007) and methyl jasmonate (Kang et al., 2004) are plant defense hormones that stimulate protective plant secondary metabolites in response to fungal invasion (Swanty, 2000). Both hormones have been shown to stimulate plant Taxol production when added to suspension cultures of *Taxus* plant cells (Nims et al., 2006; Wang et al., 2007). Therefore, we hypothesized that these hormones may stimulate fungal Taxol production. Salicylic acid increased fungal Taxol production ∼twofold (*P* < 0.01), whereas no significant increase was observed with methyl jasmonate (Figure 1F; Table 1).

Previously, benzoic acid was found to increase plant Taxol production by fivefold (Arthur et al., 1994) and caused restoration of Taxol biosynthesis in fungal cultures that had lost this ability following successive re-culturing (Li et al., 1998b). Benzoic acid was suggested to be a precursor of the plant Taxol side-chain (Arthur et al., 1994), though two radiolabeling studies contradicted this interpretation (Stierle et al., 1993; Li et al., 1998b); thus the exact mechanism by which benzoic acid increases Taxol production has been unclear. Given the salicylic acid result, we noted that salicylic acid differs from benzoic acid by only a single hydroxyl group at the ortho position (Figures 1F,G). Furthermore, both compounds have been shown to have direct antifungal activity on *in vitro* cultured wood-inhabiting fungus *Eutypa lata* (Amborabé et al., 2002). We found that addition of benzoic acid to a non-attenuated culture resulted in a significant increase in fungal Taxol production, by 1.7-fold (Figure 1G; *P* < 0.01, Table 1). We hypothesize that benzoic acid may be mimicking plant-derived salicylic acid.

Based on the habitat of *Paraconiothyrium* SSM001 in plant vascular tissues, next we tested the effects of two plant hormones likely localized to such tissues, strigolactone, and auxin (IAA). Strigolactones have classically been shown to facilitate plant-fungal communication by stimulating beneficial soil mycorrhizae fungal spore germination and hyphal penetration into host plant tissues (Akiyama and Hayashi, 2006; Yoneyama et al., 2007). More recently, strigolactone has been implicated as a hormone required for plant development, and strigolactone biosynthesis genes have been localized to plant vascular cells (Booker et al., 2005; Brewer et al., 2009). The addition of strigolactone did not increase fungal Taxol yield, but rather caused a dose-dependent decline (Figure 1H; Table 1) without affecting fungal mass (data not shown). We then tested auxin as it is an important hormone required for plant vascular cell formation, including wood medullary ray biogenesis (Mattsson et al., 1999). Auxin has also been reported to stimulate conidial germination and fungal growth (Nakamura et al., 1985). Application of IAA did not increase fungal Taxol, but rather caused dramatic declines (Figure 1I, Table 1).

In conclusion, fungal Taxol increased ∼20–30-fold in response to *Taxus* or pine bark extract. The increase in fungal Taxol was not due to plant taxane precursors, phytoalexin, or plant hormones with the exception of ∼ twofold increases by salicylic acid and its related compound, benzoic acid.

### Co-habitating fungi stimulate taxol production from *Paraconiothyrium* SSM001

**Figure 2:**
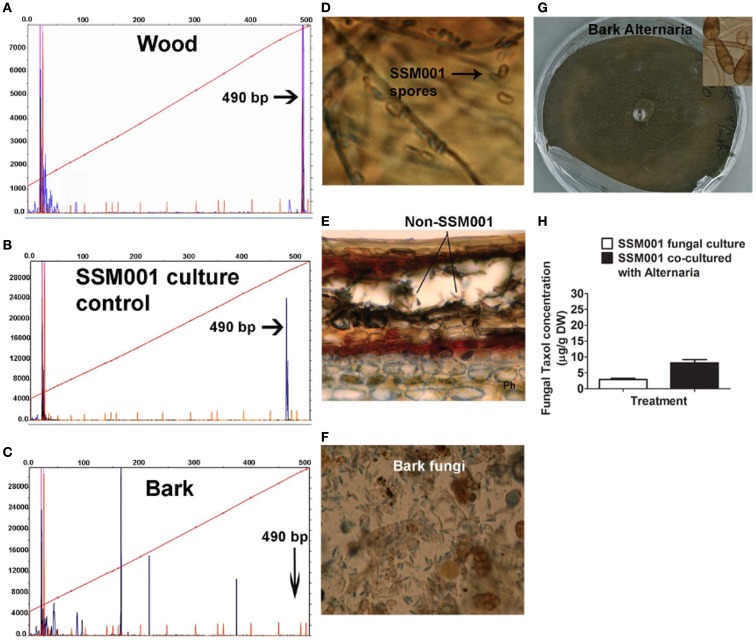
**Co-habitating fungi present in *Taxus x media* outer bark stimulate Taxol production from *Paraconiothyrium* SSM001 fungi**. **(A)** DNA fingerprinting (fungal 18S tRFLP) of *Taxus* wood (excluding bark). The *x*-axis reflects the fragment size following *Hae*III digestion. The dendrogram shows one peak (blue) corresponding to SSM001 along with other very minor peaks. **(B)** Control tRFLP of pure cultured *Paraconiothyrium* SSM001 showing a peak at 490 nucleotides. **(C)** DNA fingerprinting (fungal 18S tRFLP) of *Taxus* outer bark. The dendrogram shows five novel peaks (blue) other than SSM001. **(D)** SSM001 spores. **(E)** Non-SSM001 fungal spores in the outer bark in a transverse section of a *Taxus* branch. **(F)** Close-up of spores from *Taxus* outer bark, stained with Trypan blue, showing the diversity of fungi. **(G)** Isolation and culturing of pure *Alternaria* fungus from the outer bark of *T. x media*. **(H)** Effect of co-culturing of pure *Alternaria* fungus with SSM001 in liquid culture.

Since none of the candidate plant elicitors caused a dramatic increase in fungal Taxol production, then given the fungicidal properties of Taxol (Young et al., 1992), we then hypothesized that other fungi contained within plant bark and wood may be the apparent major wood/bark elicitors of fungal Taxol production. We also hypothesized that if invasive fungi are the major elicitors of fungal Taxol production, then the reason that *Taxus* and pine wood had much less dramatic effects than bark on eliciting Taxol production (Table 1) may be that wood harbors a relatively small amount of non-*Paraconiothyrium* species. First we tested whether *Taxus* wood contains fungi other than the Taxol-producing *Paraconiothyrium* SSM001 strain. DNA isolated from *Taxus* wood and subjected to 18S rDNA terminal restriction fragment length polymorphism (tRFLP) analysis showed only one peak at 490 bp corresponding to *Paraconiothyrium* SSM001, along with two very minor peaks (Figure 2A) compared to pure *Paraconiothyrium* tRFLP (Figure 2B). In contrast, DNA isolated from *Taxus* bark and subjected to 18S rDNA tRFLP analysis showed the presence of >5 fungal peaks, none of which were *Paraconiothyrium* SSM001 (Figure 2C) which was shown to have a peak at ∼490 nucleotides (Figure 2B). Compared to pure *Paraconiothyrium* SSM001 spores (Figure 2D), a transverse section of a *Taxus* branch showed the presence of non-*Paraconiothyrium* spores in the outer bark (Figures 2E,F). To identify the TRFLP peaks and spore types, spores were germinated, and genomic DNA was isolated from mycelia, which was then sequenced using 18S rDNA primers (Table 2). Consistent with the earlier data, only one unique endophyte was identified in *Taxus* wood, though it was a *Paraconiothyrium* species. In contrast, six unique fungi were identified in bark, including species belonging to *Alternaria*, *Bionectria*, *Aspergillus*, *Diplodia*, *Rosellinia*, and *Pestalotiopsis* (Table 2).

**Table 2:** **Sequences of fungal isolates from *Taxus* tissues and their taxonomic identities**.

Tissue	Fungal isolate 18S rDNA	Fungal species	Identity value (%)
Wood	CGAGTTTTTCNGCAAGANTTAGCATGGAATGGAATAGGACGTGCGGTCCTATTTTGTTGGTTTCTAGGACCGCCGTAATGATTAATAGGGACAGTCGGGGGCATCAGTATTCAATTGTCAGAGGTGAAATTCTTGGATTTATTGAAGACTAACTACTGCGAAAGCATTTGCCAAGGATGTTTTCATTAATCAGTGAACGAAAGTTAGGGGATCGAAGACGATCAGATACCGTCGTAGTCTTAACCATAAACTATGCCGACTAGGGATCGGGCGGTGTTTCTATTGTGACCCGCTCGGCACCTTACGAGAAATCAAAGTGTTTGGGTTCTGGGGGGAGTATGGTCGCAAGGCTGAAACTTAAAGAAATTGACGGAAGGG CACCACCAGGCGTGGAGCCTGCGGCTTAATTTGACTCAACACGGGGAAACTCACCAGGTCCAGA	*Paraconiothyrium variabile*	99
Bark	TGGACCTGGNGAGTTTCCCCGTGTTGAGTCNANNNNNNNNNNNNNNTCCACGCCTGGTGGTGACCTTCCGTCAATTTCTTTAAGTTTCAGCCTTGCGACCATAATCCCCCCAGAACCCAAAAACTTTGATTTCTCGTAAGGTGCCGAGCGAGTCAGAAAAAGAACATCGCCCGATCCCTAGTCGGCATAGTTTACGGTTAAGACTACGACGGTATCTGATCGTCTTCGATCCCCTAACTTTCGTTCACTGATTAATGAAAACATCCTTGGCAAATGCTTTCGCAGTAGTTAGTCTTCAGTAAATCCAAGAATTTCACCTCTGACAACTGAATACTGATGCCCCCGACTGTTCCTGTTAATCATTGCGGCGTCTCTAGAAACCAACAAAATAGAAACGCACGTCCTATTTCATTCCATGCTAANTCTTT	*Alternaria alternata*	99
Bark	AAGTAAAAGTCGTAACAAGGTCTCCGTTGGTGAACCAGCGGAGGGATCATTACCGAGTTTACAACTCCCAAACCCATGTGAACATACCTACTGTTGCTTCGGCGGGATTGCCCCGGGCGCCTCGTGTGCCCCGGATCAGGCGCCCGCCTAGGAAACTTAATTCTTGTTTTATTTTGGAATCTTCTGAGTAGTTTTTACAAATAAATAAAAACTTTCAACAACGGATCTCTTGGTTCTGGCATCGATGAAGAACGCAGCGAAATGCGATAAGTAATGTGAATTGCAGAATTCAGTGAATCATCGAATCTTTGAACGCACATTGCGCCCGCCAGTATTCTGGCGGGCATGCCTGTCTGAGCGTCATTTCAACCCTCATGCCCCTAGGGCGTGGTGTTGGGGATCGGCCAAAGCCCGCGAGGGACGGCCGGCCCCTAAATCTAGTGGCGGACCCGTCGTGGCCTCCTCTGCGAAGTAGTGATATTCCGCATCGGAGAGCGACGAGCCCCTGCCGTTAAACCCCCAACTTTCCAAGGTTGACCTCAGATCAGGTAGGAATACCCGCTGAACTTAAGCAT	*Bionectria* spp	100
Bark	AGAGGAAGTAAAAGTCGTAACAAGGTTTCCGTAGGTGAACCTGCGGAAGGATCATTACCGAGTGTAGGGTTCCTAGCGAGCCCAACCTCCCACCCGTGTTTACTGTACCTTAGTTGCTTCGGCGGGCCCGCCATTCATGGCCGCCGGGGGCTCTCAGCCCCGGGCCCGCGCCCGCCGGAGACACCACGAACTCTGTCTGATCTAGTGAAGTCTGAGTTGATTGTATCGCAATCAGTTAAAACTTTCAACAATGGATCTCTTGGTTCCGGCATCGATGAAGAACGCAGCGAAATGCGATAACTAGTGTGAATTGCAGAATTCCGTGAATCATCGAGTCTTTGAACGCACATTGCGCCCCCTGGTATTCCGGGGGGCATGCCTGTCCGAGCGTCATTGCTGCCCATCAAGCACGGCTTGTGTGTTGGGTCGTCGTCCCCTCTCCGGGGGGGACGGGCCCCAAAGGCAGCGGCGGCACCGCGTCCGATCCTCGAGCGTATGGGGCTTTGTCACCCGCTCTGTAGGCCCGGCCGGCGCTTGCCGAACGCAAATCAATCTTTTTCCAGGTTGACCTCGGATCAGGTAGGGATACCCGCTGAACTTAAGCATA	*Aspergillus* spp	100
Bark	AGTcGTAACAAGGTTTCcGTAGGTGAACCTGCGGAAGGATCATTACCGAGTTCTCGGGCTTCGGCTCGAATCTCCCACCCTTTGTGAACATACCTCTGTTGCTTTGGCGGCTCTTTGCCGCGAGGAGGCCCTCGCGGGCCCCCCCGCGCGCTTTCCGCCAGAGGACCTTCAAACTCCAGTCAGTAAACGTCGACGTCTGATAAACAAGTTAATAAACTAAAACTTTCAACAACGGATCTCTTGGTTCTGGCATCGATGAAGAACGCAGCGAAATGCGATAAGTAATGTGAATTGCAGAATTCAGTGAATCATCGAATCTTTGAACGCACATTGCGCCCCCTGGCATTCCGGGGGGCATGCCTGTTCGAGCGTCATTACAACCCTCAAGCTCTGCTTGGTATTGGGCGCCGTCCTCTCTGCGGACGCGCCTTAAAGACCTCGGCGGTGGCTGTTCAGCCCTCAAGCGTAGTAGAATACACCTCGCTTTGGAGCGGTTGGCGTCGCCCGCCGGACGAACCTTCTGAACTTTTCTCAAGGTTGACCTCGGATCA	*Diplodia* spp	99
Bark	GAAGTAAAAGTCGTAACAAGGTCTCCGTTGGTGAACCAGCGGAGGGATCATTAGAGAGTGCCCTACTCCCAAAACCCATGTGAACTTACCTGTACGTTGCCTCGGCGGGGGAGGGGCTGGCCACCCCCCCTCCGCCAGGCGGCCCACCAAACCCTGTTTAGCCCTGAATCTCTGAGACGATAAAACAATGAGTTAAAACTTTCAACAACGGATCTCTTGGCTCTGGCATCGATGAAGAACGCAGCGAAATGCGATACGTAGTGTGAATTGCAGAATTTAGTGAATCATCGAATCTTTGAACGCACATTGCGCCCGCCAGTATTCTGGCGGGCATGCCTGTTCGAGCGTCATTTCAACCCCTTAAGCCCTTGTCGCTTAGTGTTGGGAGCCGACGGCGTCCTGCCGTCGCTCCTCAAATCCAGTGGCGGAGCCGGTTTCGCGCTCTGGGCGTAGTAGATTTTCTCCATCTCGCCTGCAGCCGGGGCCGGCCTCCCTGCCGTAAAACCACCACCAATGTACCCAAAGGTTGACCTCGAATCAGGTAGGAATACCCGCTGAACTTAAGCATAT	*Rosellinia* spp	99
Bark	TTTAGGTGANACTATAGAATACAGCGGCCGCGAGCTCGGGCCCCCACACGTGTNNNNNNNAGNNANCCTAGGCTCGAGAAGCTTGTCGACGAATTCAGATTTCTGGACCTGGTGAGTTTCCCCGTGTTGAGTCAAATTAAGCCGCAGGCTCCACCCCTGGTGGTGCCCTTCCGTCAATTTCTTTAAGTTTCAGCCTTGCGACCATACTCCCCCCAGAACCCAAAGACTTTGATTTCTCGTAAGGTGCCGAACGGGTCAATAAGTAACACCGTCCGATCCCTAGTCGGCATAGTTTATGGTTAAGACTACGACGGTATCTGATCGTCTTCGATCCCCTAACTTTCGTTCCTGATTAATGAAAACATCCTTGGCAAATGCTTTCGCAGTAGTTAGTCTTCAATAAATCCAAGAATTTCACCTCTGACAATTGAATACTGATGCCCCGACTGTCCCTATTAATCATTACGGCGGTCCTAGAAACCAACAAAATAGAACCACACGTCCTATTTCATTCCATGCTAAAATCACGAATTCTGGATCCGATACGTAACGCGTCTGCAGCATGCGTGGTACCGAGCTTTCCCTATAGTGAGTCGTATTAGAGCTTGGCGTAATCATGGTCATAGCTGTTTCCTGTGTGAAATTGTTATCCGCTCACAATTCCACACAACATACGAGCCGGAAGCATAAAGTGTAAAGCCTGNGGTGCCTAATGAGTGAGCTAACTCACATTAATTGCGTTGCGCTCACTGCCCGCTTTCCAGTCGGGAAACCTGTCGTGCCAGCTGCATTAATGAATCGGCCAACGCGCGGGGAGAGGCGGTTTGCGTATTGGGCGCTCTTCCGCTTCCTCGCTCACTGANTCGCTGCGCTCGGTCGTTCGGCTGCGGCGAGCGGTATCAGCTCACTCNAAGGCGGTA	*Pestalotiopsis* spp	99
Needles	TAGGTGANNCTATAGAATACAGCGGCCGCGAGCTCGGGCCCCCACACGTGTGGTCTAGAGCTAGCCTAGGCTCGAGAAGCTTGTCGACGAATTCAGATTTTAGCATGGAATAGAATAGGACGTGTGGTTCTATTTTGTTGGTTTCTAGGACCGCCGTAATGATTAATAGGGATAGTCGGGGGCGTCAGTATTCAGCTGTCAGAGGTGAAATTCTTGGATTTGCTGAAGACTAACTACTGCGAAAGCATTCGCCAAGGATGTTTTCATTAATCAGGGAACGAAAGTTAGGGGATCGAAGACGATCAGATACCGTCGTAGTCTTAACCATAAACTATGCCGACTAGGGATCGGACGGTGTTTCTATTATGACCCGTTCGGCACCTTACGAGAAATCAAAGTTTTTGGGTTCTGGGGGGAGTATGGTCGCAAGGCTGAAACTTAAAGAAATTGACGGAAGGGCACCACAAGGCGTGGAGCCTGCGGCTTAATTTGACTCAACACGGGGAAACTCACCAGGTCCAGAAATCACGAATTCTGG	*Paecilomyces variotii*	99
Needles	TTTAGGTGANNCTATAGAATACAGCGGCCGCGAGCTCGGGCCCCCACACGTGTGNNNTANAGCTAGCCTAGGCTCGAGAAGCTTGTCGACGAATTCAGATTTTAGCATGGAATAAAATAGGACGTCGCGGTTCTATTTTGTTGGTTTCTAGGACCGCCGTAATGATTAATAGGGACAGTCGGGGGCATCAGTATTCAATCGTCAGAGGTGAAATTCTTGGATCGATTGAAGACTAACTACTGCGAAAGCATTTGCCAAGGATGTTTTCATTAATCAGGAACGAAAGTTAGGGGATCGAAAACGATCAGATACCGTTGTAGTCTTAACCATAAACTATGCCGACTAGGGATCGGGCGGTGTTATTTCTTGACCCGCTCGGCACCTTACACGAAAGTAAAGTTTTTGGGTTCTGGGGGGAGTATGGTCGCAAGGCTGAAACTTAAAGAAATTGACGGAAGGGCACCACAAGGGGTTAACGTTATTGTTGCACGCAGACTCTGCCCCAGAAAGCAGCCTACGCAAGTAGGGTGTGGTGCCCTTTATATGCTAGTCGGCCGGAAGGCCGGCGATACCTTCAAATTGCGGGGATAGCCTTAGAGGCCCTGGTACCAAGCACCACTCCGAAAGGTTGGTGTGGCGTAGCTAATCACTACGGTACGGTAATAATCCAGGTGCATTGGCCGATCCGCAGGCAAGCCCCTCTGGGCCCCCTCGGGGGCCCCCTGTGGGAAGCTTCAGAGACTAAACGGAGGTAGGTCTGTTCGGGGAAACCCATACAGGCTTAAGATATAGTCCGAGCCAGCCCTGAAAAGGGCTGGGANGGTTGCCTTAACAAGCGCCTGANAAATGGGAGCCTGCGGCTTAATTTGACTCNACACGGGGAAACTCACCAGGTCCAGAAATCACGAATTCTGGATCCGATACGTAAC	*Phomopsis* spp	99
Needles	TTAGGTGANNCTATAGAATACAGCGGCCGCGAGCTCGGGCCCCCACACGTGTGGTCTAGAGCTAGCCTAGGCTCGAGAAGCTTGTCGACGAATTCAGATTTTAGCATGGAATAGAATAGGACGTGTGGTTCTATTTTGTTGGTTTCTAGGACCGCCGTAATGATTAATAGGGATAGTCGGGGGCGTCAGTATTCAGCTGTCAGAGGTGAAATTCTTGGATTTGCTGAAGACTAACTACTGCGAAAGCATTCGCCAAGGATGTTTTCATTAATCAGGGAACGAAAGTTAGGGGATCGAAGACGATCAGATACCGTCGTAGTCTTAACCATAAACTATGCCGACTAGGGATCGGACGGTGTTTCTATTATGACCCGTTCGGCACCTTACGAGAAATCAAAGTTTTTGGGTTCTGGGGGGAGTATGGTCGCAAGGCTGAAACTTAAAGAAATTGACGGAAGGGCACCACAAGGCGTGGAGCCTGCGGCTTAATTTGACTCAACACGGGGAAACTCACCAGGTCCAGAAATCACGAATTCTGGATCCGATACGTAACGCGTCTGCAGCATGCGTGGTACCGAGCTTTCCCTATAGTGAGTCGTATTAGAGCTTGGCGTAATCATGGTCATAGCTGTTTCCTGTGTGAAATTGTTATCCGCTCACAATTCCACACAACATACGAGCCGGAAGCATAAAGTGTAAAGCCTGGGGTGCCTAATGAGTGAGCTAACTCACATTAATTGCGTTGCGCTCACTGCCCGCTTTCCAGTCGGGAAACCTGTCGTGNCAGCTGCATTAATGAATCGGCCAACGCGCGGGGAGAGGCGGTTTGCGTATTGGGCGCTCTTCCGCTTCCTCGCTCACTGACTCGCTGCGCTCGGTCGTTCGGCTGCGGCGAGCGGNNATCAGCTCACTCNAAGGCGGTAATAC	*Aspergillus* spp	99
Seeds	TTTAGGTGANACTATAGAATACAGCGGCCGCGAGCTCGGGCCCCCACACGTGTGGTCTAGAGCTAGCCTAGGCTCGAGAAGCTTGTCGACGAATTCAGATTTTAGCATGGAATTAAATAGGACGTGTGGTCCTATTTTGTTGGTTTCTAGGACCGCCGTAATGATTAATAGGGACAGTCGGGGGCATCAGTATTCAATTGTCAGAGGTGAAATTCTTGGATTTATTGAAGACTAACTACTGCGAAAGCATTTGCCAAGGATGTTTTCATTAATCAGTGAACGAAAGTTAGGGGATCGAAGACGATCAGATACCGTCGTAGTCTTAACCGTAAACTATGCCGACTAGGGATCGGGCGATGTTCTTTTTCTGACTCGCTCGGCACCTTACGAGAAATCAAAGTTTTTGGGTTCTGGGGGGAGTATGGTCGCAAGGCTGAAACTTAAAGAAATTGACGGAAGGGCACCACCAGGCGTGGAGCCTGCGGCTTAATTTGACTCAACACGGGGAAACTCACCAGGTCCAGAA	*Phoma* spp	99

One of the resident bark fungi (Figure 2G) identified as an *Alternaria* species by spore morphology (Figure 2G, inset) and 18S rDNA sequencing (Table 2) was co-cultured with *Paraconiothyrium* SSM001. Alone, cultures of *Alternaria* did not show any Taxol production using TLC or Taxol immunoassays (data not shown). However, *Alternaria* co-culturing caused a significant, 2.7-fold increase in *Paraconiothyrium* Taxol production compared to the control (Figure 2H; *P* < 0.01, Table 1).

**Figure 3:**
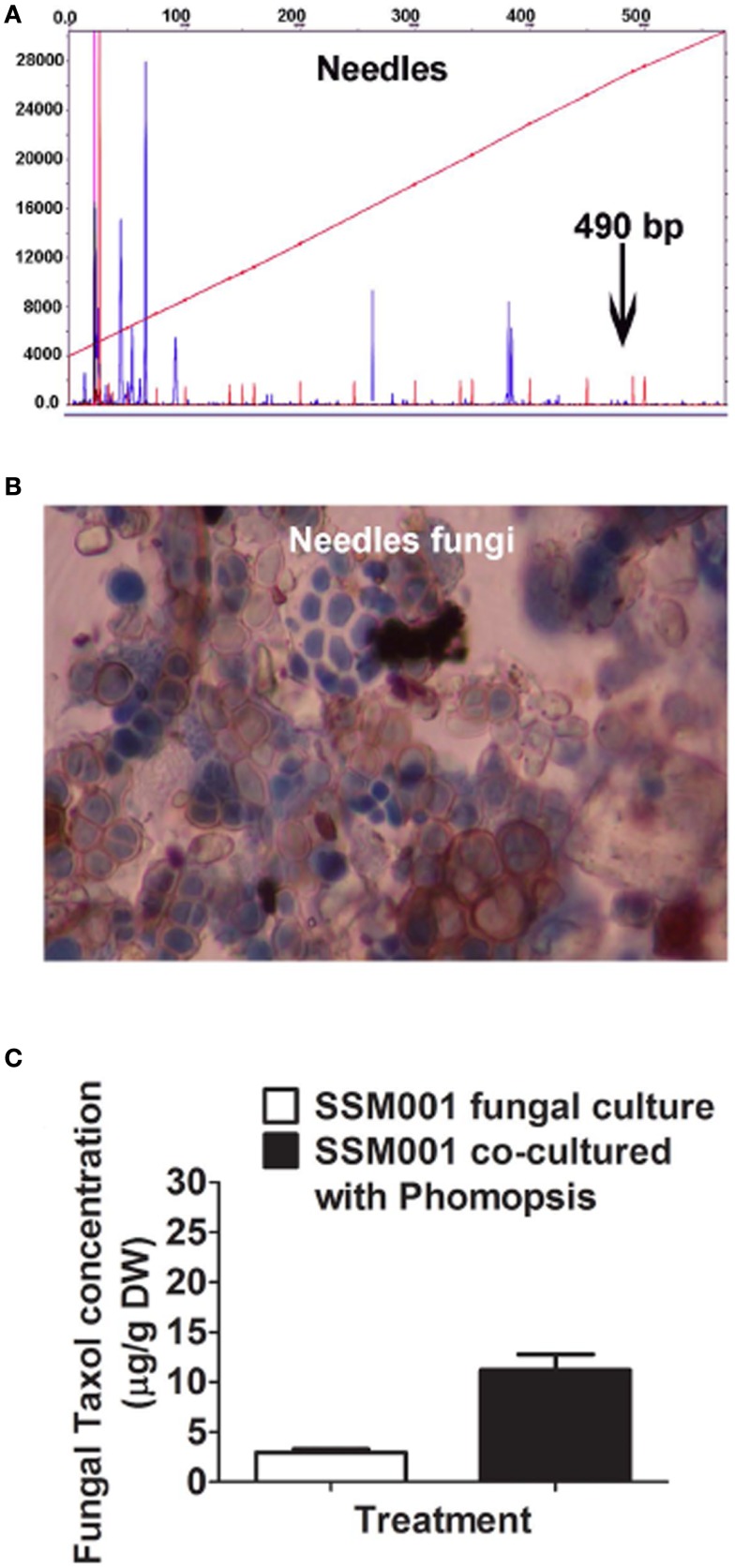
**Co-habitating fungi present in *Taxus x media* needles stimulate Taxol production from *Paraconiothyrium* SSM001 fungi**. **(A)** tRFLP analysis of DNA pooled from *Taxus* needles showing several fungal peaks, none of which corresponded to SSM001. **(B)** Detection of different fungal spores from *T. x media* needles. **(C)** Effect of co-culturing of pure *Phomopsis* fungus isolated from *Taxus* needles with SSM001 in liquid culture.

Since it was previously shown that aqueous yew needle extract can elicit fungal Taxol production ≤100-fold, we also asked whether yew needles were a reservoir of non-*Paraconiothyrium* fungi. In *T. x media* needles, we found the presence of ∼5–7 distinct fungal types based on tRFLP analysis (Figure 3A) and spore morphology (Figure 3B). Culturing of the spores, followed by 18S rDNA amplification and sequencing from hyphae revealed three distinct fungi belonging to the genera identified as *Paecilomyces*, *Phomopsis*, and *Aspergillus* (Table 2). Co-culturing *Paraconiothyrium* SSM001 with one needle isolate, *Phomopsis*, showed a significant, 3.8-fold increase in fungal Taxol (Figure 3C; *P* < 0.01, Table 1). Cultures of *Phomopsis* alone did not show any Taxol production using TLC or Taxol immunoassays (data not shown).

**Figure 4:**
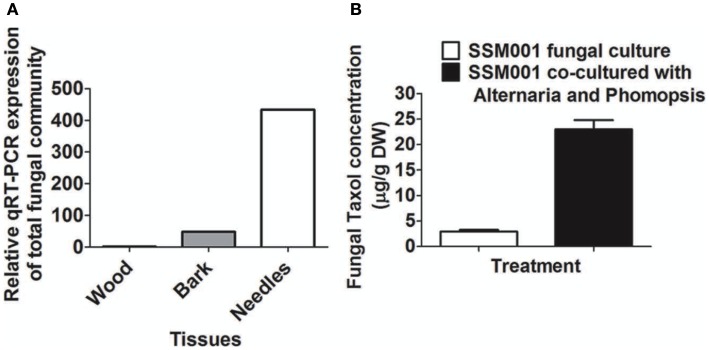
**Expression ratio of total fungal community in *Taxus* needles in comparison to *Taxus* bark and wood**. **(A)** Expression ratio of total fungal community in *Taxus* needles and bark in comparison to *Taxus* wood. **(B)** Effect of co-culturing of *Alternaria* and *Phomopsis* fungi isolated from outer bark and needles, respectively on SSM001 Taxol production in liquid culture.

Quantitative real-time PCR using specific fungal primers showed 400-fold higher concentrations of fungi in *Taxus* needles than in wood and 100-fold more in needles than in bark (Figure 4A). These results are consistent with at least some of the Taxol elicitor activity of *Taxus* needles (Stierle et al., 1995) coming from resident fungi.

To determine whether resident fungi can act in an additive manner to elicit fungal Taxol production, SSM001 was co-cultured with *Alternaria* isolated from *Taxus* bark and *Phomopsis* isolated from *Taxus* needles; co-culturing caused a 7.8-fold increase in fungal Taxol (Figure 4B; *P* < 0.01, Table 1) suggesting a synergistic effect. The data suggest that, in combination with plant-derived factors, resident fungi within *Taxus* bark (and potentially other tissues) contribute to the strong elicitor activity of this tissue.

## Discussion

This study has used knowledge of the habitat (Figure 1A; Table 2) of a Taxol-producing fungus *Paraconiothyrium* SSM001 to systematically predict and test elicitors of fungal Taxol. We found that addition of extracts from the habitat of *Paraconiothyrium* SSM001, namely from wood and bark, caused ∼10–30-fold increases in Taxol synthesis (Figures 1B,C), consistent with a previous study in which *Taxus* needle extracts increased fungal Taxol production by 100-fold (Stierle et al., 1995). Testing of candidate plant elicitors showed that the plant defense hormone salicylic acid, and its related compound, benzoic acid, caused twofold increases in fungal Taxol (Figure 1). A more important elicitor, however, was one of several resident non-*Paraconiothyrium* fungi living within elicitor outer bark (Figures 2 and 4; Table 2) suggesting that signals or other compounds released from resident pathogenic and endophytic fungi, present in extracts of bark, might contribute to the active “plant”-derived elicitor(s) of fungal Taxol, including from *Taxus* needles (Figure 3).

### Conifer wood and bark extracts stimulate fungal taxol production

Since *Taxus* and pine tissue extracts have been shown here (Figure 1) and elsewhere (Stierle et al., 1995) to stimulate fungal Taxol production, and since some experiments with reported Taxol-producing fungi show no authentic Taxol production (Staniek et al., 2009), it has been suggested that fungi require the yew plant to synthesize Taxol. Co-culture of a Taxol-producing fungus, *F. mairei*, with *T. chinensis*, was previously shown to elicit fungal Taxol production 38-fold, but the elicitor mechanism was not tested (Li et al., 2009). We found no evidence that *Taxus* contributes precursors to fungal Taxol, as we observed no increase in Taxol accumulation when pure *Paraconiothyrium* was co-cultured with a *Taxus* suspension line producing taxane (Figure 1). Thus, we used knowledge of the fungal habitat in *Taxus* wood vascular tissues (Figure 1A) to test other plant factors. The plant hormones, strigolactone and IAA (auxin), appeared to be good candidates for a fungal Taxol elicitor since both are associated with plant vascular cells (Mattsson et al., 1999; Booker et al., 2005; Brewer et al., 2009) and both have been shown to stimulate fungal germination and growth (Nakamura et al., 1985). Surprisingly, we found that both plant hormones inhibited fungal Taxol production (Figures 1H,I). As one report had suggested that IAA can have fungicide activity (Yu et al., 2008), we tested it but observed no decrease in fungal growth (data not shown). As plants respond to fungi by producing defense compounds and hormones, we also tested resveratrol, a plant defense compound shown to be induced in conifers in response to pathogenic fungi (Evensen et al., 2000). However, we found that resveratrol strongly inhibited fungal Taxol production (Figure 1E). Resveratrol has been shown to act as a fungicide against a variety of wood decaying fungi (Seppänen et al., 2004; Välimaa et al., 2007) which might explain this result. We did, however, identify salicylic acid, an important plant defense hormone, as causing a significant, twofold increase in fungal Taxol production in the absence of any plant tissues (Figure 1F; Table 1). Interestingly, salicylic acid was previously shown to increase Taxol production in *Taxus* suspension cultures (Wang et al., 2004, 2007). Though limited information exists on the direct effects of salicylic acid on fungi *in vitro*, salicylic acid has been shown to induce mycotoxin production in *F. oxysporum* f. sp. niveum cultured *in vitro* (Wu et al., 2008). Salicylic acid has also been shown to inhibit mycelia growth, conidiation, and spores in several *in vitro* cultured fungi including *Saccharomyces cerevisiae*, *Sphaerotheca fuliginea*, *Sclerotium* spp, and *F. oxysporum* (Amborabé et al., 2002; Wu et al., 2008). Finally, we tested the effect of benzoic acid which was previously hypothesized to either restore silenced fungal Taxol production (Li et al., 1998b) or act as a precursor of the Taxol side-chain (Arthur et al., 1994), though radiolabeling experiments did not support the latter hypothesis (Stierle et al., 1993; Li et al., 1998b). Our results suggest a new hypothesis, that benzoic acid in fact may be mimicking the elicitor effects of the related compound, salicylic acid, which would be expected to be produced by *Taxus* in response to fungal colonization. Salicylic acid and benzoic acid differ by a single hydroxyl group at the ortho position of the benzene ring (Amborabé et al., 2002).

Therefore, of the 10- to 30-fold increases caused by plant extracts on fungal Taxol production (Figures 1–3; Stierle et al., 1995), we identified only one plant-derived elicitor that caused a twofold increase. Careful fractionation of *Taxus* extracts may be required to identify additional active plant-derived elicitors of fungal Taxol production.

### Fungi resident in *Taxus* tissues stimulate taxol production from *Paraconiothyrium*

We tested the effects of co-culturing only one of the several fungal species found in *Taxus* bark and discovered that it stimulated *Paraconiothyrium* SSM001 Taxol production 2.7-fold (Figure 2; Table 1). Subsequent co-culturing of two fungi inhabiting *Taxus* was shown to increase Taxol production from SSM001 by 7.8-fold (Figure 4; Table 1). We thus hypothesize that the 10- to 30-fold increase in fungal Taxol caused by *Taxus* and pine wood and bark may in fact be due to multiple pathogenic and non-pathogenic fungi contained within these tissues acting together as elicitors. As to why bark and needles were observed to act as greater elicitors than wood of fungal Taxol production, one possibility is that wood has a low titer of fungi. Consistent with this hypothesis, our tRFLP analysis and direct 18S rDNA sequencing of cultured spores demonstrated that *Taxus* bark and needles have several non-*Paraconiothyrium* fungi compared to wood (Figures 2 and 3; Table 2). Furthermore, quantitative real-time PCR showed 40-and 400-fold fungal quantity in bark and needles, respectively in comparison to wood.

Our results, therefore, suggest that interactions occur between different fungal species inhabiting *Taxus* that result in increased fungal Taxol biosynthesis. Prior results showed the presence of several fungi that inhabit *Taxus* plants (Farr and Rossman, 2006) but these were not tested for their effects on endophytic Taxol production. However, Taxol-producing endophytes have previously been shown to inhibit growth of non-WDF plant pathogens, including *Pythium*, *Aphanomyces*, and *Phytophthora* (Wang et al., 2000; Strobel, 2002, 2003; Strobel and Daisy, 2003). Our result is also consistent with previous studies showing that fungal metabolism can be affected by the metabolites of other fungi (Gayed, 1963). In the future, fractionation based bioassays using the fungi identified here as stimulating fungal Taxol production, may be used to identify candidate diffusible metabolites that affect fungal Taxol biosynthesis. It will then be useful to determine whether these compounds are also present in *Taxus* bark alcoholic extracts as hypothesized in this manuscript.

A greater number of previous studies have focused on fungal-plant interactions than fungal-fungal interactions within plants. For example, several studies showed that fungi or fungal metabolites such as chitin can elicit plant Taxol, though the mechanism was not investigated (Wang et al., 2001; Li and Tao, 2009; Zhang et al., 2011). Endophytic fungi have also been shown to stimulate rate-limiting enzymes in the terpenoid pathway, namely 3-hydroxy-3-methylglutaryl coenzyme A reductase (HMGR) and 1-deoxy-d-xylulose 5-phosphate synthase (DXR; Gao et al., 2011). Arbuscular mycorrhizal (AM) fungi have also been shown to stimulate biosynthesis of plant terpenoids, alkaloids, and phenolics (Zhao and Yan, 2006). In this context, in the future, it will also be interesting to explore the possibility that compounds derived from endophytic fungi or other resident fungi may be directly interacting with the plant-derived Taxol pathway.

In conclusion, this study suggests that fungal endophytes may interact with other resident fungi to stimulate secondary metabolite production. The extent of this interaction *in planta* needs to be analyzed.

## Conflict of Interest Statement

The authors declare that the research was conducted in the absence of any commercial or financial relationships that could be construed as a potential conflict of interest.
